# Secondary Angle Closure Caused by Anterior Displacement of Capsular Tension Ring and Intraocular Lens Due to Aqueous Misdirection

**DOI:** 10.7759/cureus.55716

**Published:** 2024-03-07

**Authors:** Kensuke Goto, Ryo Tomita, Jiro Hiraiwa, Mitsuki Kawabe, Koji M Nishiguchi, Kenya Yuki

**Affiliations:** 1 Department of Ophthalmology, Konan Kosei Hospital, Konan, JPN; 2 Department of Ophthalmology, Nagoya University Graduate School of Medicine, Nagoya, JPN

**Keywords:** anterior segment optical coherence tomography, secondary angle closure, anterior displacement, capsular tension ring, acute angle closure crisis

## Abstract

A capsular tension ring (CTR) is used for support to stabilize the capsular bag and intraocular lens (IOL) during and after cataract surgery. Although complications involving the CTR-IOL complex are not uncommon, cases of anterior displacement leading to complications are rare. This report presents a case of secondary angle closure caused by anterior displacement of the CTR-IOL complex due to aqueous misdirection and reports unique findings noted on anterior segment optical coherence tomography (AS-OCT). The patient, a 69-year-old woman, developed an acute angle closure crisis (AACC) and underwent cataract surgery with the implantation of a CTR and IOL. Post-surgery, there was an improvement in the central depth of the anterior chamber, but the patient experienced intermittent spikes in intraocular pressure. AS-OCT revealed a flat center of the iris and a closed anterior chamber angle which are plateau-iris-like findings. Secondary angle closure was caused by the CTR-IOL complex which was anteriorly displaced and pushed the peripheral iris owing to aqueous misdirection syndrome. Three weeks after the initial surgery, the patient underwent CTR removal, anterior vitrectomy, and intrascleral lens fixation. After the second surgery, intraocular pressure was normalized without any medications, and the anterior chamber angle was enlarged. This case provides a better understanding of secondary angle closure caused by the anterior displacement of the CTR-IOL complex and highlights the importance of AS-OCT in the detection of such complications.

## Introduction

A capsular tension ring (CTR) is used for support to stabilize the capsular bag and intraocular lens (IOL) during and after cataract surgery in cases of zonular instability [1-3]. Since the use of CTRs is increasing, there have been clinical reports of CTR-IOL complex, which consists of CTR, IOL, and lens capsule, displacement toward the vitreous cavity or downward displacement, which can lead to reduced visual acuity, uveitis, and cystoid macular edema [4-6]. However, there are limited reports concerning the anterior displacement of the CTR-IOL complex [7]. Herein, we present a case of secondary angle closure caused by anterior displacement of the CTR-IOL complex following cataract surgery for acute angle closure crisis (AACC), and the unique findings that were noted on anterior segment optical coherence tomography (AS-OCT).

## Case presentation

**Figure 1:**
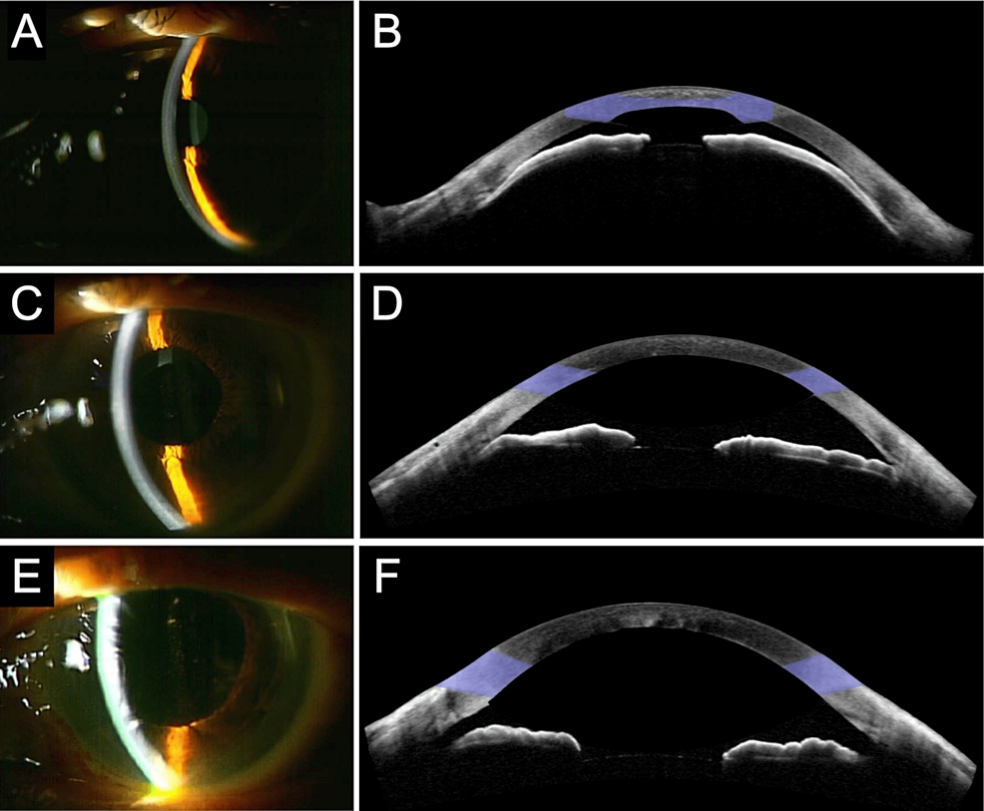
Slit-lamp photographs and AS-OCT (A, B) Images of the initial visit with AACC. The shallowing of the anterior chamber and the narrow angle are observed. (C, D) Images two days after cataract surgery for AACC with implantation of IOL and CTR. AS-OCT shows an improved anterior chamber depth, flat center of the iris, and closed anterior chamber angle which are plateau-iris like findings. (E, F) Images six days after CTR removal, anterior vitrectomy, and transscleral IOL fixation. The anterior chamber depth is further improved, and the anterior chamber angle is enlarged. AACC = acute angle closure crisis, AS-OCT = anterior segment optical coherence tomography, IOL = intraocular lens, CTR = capsular tension ring.

A 69-year-old woman with no history of ophthalmologic disease visited the emergency room with a sudden onset of eye pain and vision loss in her left eye. Initial examination revealed a shallowed anterior chamber and corneal edema. Her visual acuity was 20/400, and her intraocular pressure (IOP) was alarmingly high at 66 mmHg. She was diagnosed as having AACC due to pupillary block and was treated with 2% pilocarpine eye drops, intravenous mannitol with 300 mL of 20% injection over 30 minutes soon after the diagnosis, and laser iridotomy. After the initial treatment, the AACC resolved and the IOP decreased to 15 mmHg (Figures 1A, 1B). Fundus examination and OCT fundus images showed no abnormalities. The axial length was 23.2 mm in the right eye and 23.1 mm in the left eye. Spherical equivalent refraction was -1.5 diopters in the right eye. The left eye’s refraction was not measurable due to corneal Descemet membrane folds and anterior chamber inflammation. The patient underwent cataract surgery on the left eye two days after the initial visit. The procedure was performed under sub-Tenon’s anesthesia with 2% lidocaine. Zonular weakness and severe phacodonesis were observed during capsulorhexis using capsulorhexis forceps. After hydrodissection, a CTR of 13.0 mm in diameter and 0.18 mm in thickness (HOYA CTR; HOYA Co., Tokyo, Japan) was inserted using an injector to stabilize the lens capsule. Ultrasound phacoemulsification aspiration was performed, and a three-piece IOL of 13.0 mm in diameter (Avansee PN6AS, Kowa Co., Ltd., Nagoya, Japan) was inserted into the capsular bag without any complications.

The day following the surgery, there was an improvement in the central depth of the anterior chamber. However, the patient experienced intermittent elevations in intraocular pressure (IOP), ranging between 15 and 50 mmHg. AS-OCT findings showed an anterior chamber depth of 2.3 mm, a flat center of the iris, and a closed anterior chamber angle which is plateau-iris-like findings (Figures 1C, 1D). In the slit-lamp gonioscopy, anterior trabecular meshwork was observable in the inferior angle, but only Schwalbe’s line was observable in other quadrants. The refractive value was myopic by -3.75 diopters from the expected value. These clinical findings led to the conclusion that the CTR-IOL complex had been anteriorly displaced due to aqueous misdirection, which was contributing to the angle closure.

The intermittent IOP elevation continued even after treatment with oral acetazolamide (250 mg twice daily), 0.4% ripasudil eye drops (twice daily), and 0.1% brimonidine tartrate eye drops (twice daily). As a result, the patient underwent a second surgery three weeks after the initial surgery to treat the aqueous misdirection. Standard 25-gauge three-port pars plana vitrectomy was performed under retrobulbar anesthesia with 2% lidocaine. The CTR-IOL complex was separated using a vitrectomy cutter and the CTR was removed through a corneal incision. Anterior vitrectomy was subsequently performed. The IOL inserted during the initial surgery was fixed with flanged haptics in the scleral tunnel.

After the second surgery, the visual acuity became 20/20, and the IOP normalized to 19 mmHg in the first postoperative week without any medications. The anterior chamber depth improved to 3.5 mm. The postoperative refractive value improved to a -0.5-diopter myopic value from the expected value. The compression of the peripheral iris resolved, and no peripheral anterior synechiae (PAS) was observed in the slit-lamp gonioscopy. The anterior chamber angle was enlarged, but plateau-iris-like findings remained, as observed on AS-OCT (Figures 1E, 1F). The IOP remained within the normal range without any medications for at least one year after surgery.

## Discussion

In cases of AACC, cataract surgery is considered the first-line treatment for lowering IOP and preventing the recurrence of pupillary block [8]. However, in patients with a history of AACC, the tensile strength of the zonules is decreased, and subluxation or displacement of the IOL is a common complication after cataract surgery.

The use of a CTR is a common practice to prevent intraoperative and postoperative complications caused by zonular weakness [9]. It expands the capsular bag and recruits and redistributes tension from existing zonules, leading to the reinforcement of areas with weak zonules and decentration of a mildly subluxated capsular bag. Despite these benefits, there have been reports of CTR-related complications such as postoperative subluxation or displacement [4].

While most CTR-IOL complex displacements occur downward toward the vitreous cavity [5,6], anterior displacement is less common. To the best of our knowledge, there has been only one report of anterior displacement of the CTR-IOL complex in eyes with pseudoexfoliation, which resulted in chronic and intermittent angle closure [7]. According to published literature, anterior displacement might be caused by postoperative aqueous misdirection and positive vitreous pressure. Literature suggests that factors such as short axial length and lax zonular fibers/pseudoexfoliation can collectively elevate the risk of aqueous misdirection syndrome [10]. Moreover, previous reports indicated that the lens capsular bag might become larger and thicker due to CTR implantation in the CTR-IOL complex which can circumferentially come into contact with the ciliary body and posterior iris [7,11]. As a result, the CTR-IOL complex can compress the ciliary space and cause resistance to aqueous humor migration from the posterior to the anterior chamber, leading to the potential risk for aqueous misdirection syndrome. In the present case, the patient had risk factors such as the implantation of the CTR-IOL complex as well as zonular weakness, which may have led to the anterior displacement of the CTR-IOL due to aqueous misdirection.

Aqueous misdirection syndrome is characterized by a shallow anterior chamber and elevated IOP [10]. However, in this case, AS-OCT revealed a flat center of the iris and a closed anterior chamber angle which are plateau-iris-like findings, which is atypical because the anterior chamber depth was preserved. The atypical findings could be attributed to the following: the large and thick CTR-IOL complex circumferentially pushed the peripheral iris anteriorly due to increased posterior chamber pressure, causing a closed anterior chamber angle, but the large diameter prevented further anterior displacement and shallow anterior chamber formation. This is the first report of a unique AS-OCT finding in eyes with anterior displacement of the CTR-IOL complex, highlighting the usefulness of AS-OCT in detecting anterior displacement of the CTR-IOL complex. Another possible cause of secondary angle closure could be PAS following angle-closure glaucoma or enlargement of Soemmering’s ring, as previously reported [12,13]. However, neither Soemmering’s ring in the CTR-IOL complex nor PAS was observed in this case. The opening of the anterior chamber angle after CTR removal suggests that the secondary angle closure observed on AS-OCT was caused by the anterior displacement of the CTR-IOL complex.

In a study by Bochmann et al., ultrasound biomicroscopy (UBM) examination revealed anterior displacement of the peripheral iris by the CTR-IOL complex in eyes with pseudoexfoliation, which first identified the mechanisms of the secondary angle closure caused by the anterior displacement of the CTR-IOL complex [7]. Because UBM uses high-frequency ultrasound to provide images with a relatively high axial resolution with a reasonable depth of penetration, it can visualize the posterior of the iris and the positional relationship between the lens and iris. Owing to the inferior penetration depth of AS-OCT compared with that of UBM, the CTR-IOL complex could not be observed by AS-OCT in this case. However, with the advantages of AS-OCT being non-invasive and widely used in recent years, our findings are valuable for clinicians in detecting secondary angle closure after cataract surgery with CTR implantation.

The treatment for aqueous misdirection syndrome aims to disrupt misdirection and restore normal aqueous flow. Although there are several ways to manage aqueous misdirection syndrome, there is no consensus in the literature regarding the optimal (medical or surgical) treatment strategy, even more so in cases of CTR-IOL complex. A high recurrence rate of aqueous misdirection has been reported for medical treatment, laser capsulotomy, or vitrectomy [14]. In this case, the CTR, which constituted a CTR-IOL complex with a thickened flat shape and large diameter, was removed, and vitrectomy was performed. These procedures presumably achieved normalization of IOP, which opened the angle and successfully corrected the directional disturbance of the aqueous humor.

## Conclusions

This case is the first to report secondary angle closure caused by anterior displacement of the CTR-IOL complex with unique AS-OCT findings. The anterior displacement, attributed to aqueous misdirection syndrome, presented atypically with plateau-iris-like findings due to the CTR-IOL complex's shape and size. These insights are crucial for clinicians in identifying secondary angle closure following cataract surgery with CTR implantation.
